# Massively Multiplexed Photonic Chip With Cross‐Channel Fusion For Harmonized Biosensing

**DOI:** 10.1002/advs.78168

**Published:** 2026-10-08

**Authors:** Ivan Saetchnikov, Elina Tcherniavskaia, Andreas Ostendorf, Anton Saetchnikov

**Affiliations:** ^1^ Radio Physics Department Belarusian State University Minsk Belarus; ^2^ Physics Department Belarusian State University Minsk Belarus; ^3^ Applied Laser Technologies Ruhr University Bochum Bochum Germany

**Keywords:** deep‐learning, domain adaptation, fusion, harmonized biosensing, multiplexing, optical microresonator, photonic chip, transformer, whispering gallery mode

## Abstract

Accurate, label‐free analysis of complex biological mixtures is limited by poor multiplexing scalability and inconsistent responses across sensor samples. We introduce a transformative sensing platform that integrates large‐scale photonic multiplexing with data‐driven analytics to overcome these constraints. Our modular, affordable glass‐chip whispering–gallery–mode (WGM) photonic architecture is designed to support simultaneous readout of up to 100 sensing channels comprising over 10 000 high‐Q microresonators, exceeding existing multiplexing capabilities by more than two orders of magnitude. The photonic system is reinforced by the novel, cross‐platform, deep learning framework BioCCF. It applies cross‐channel fusion in biosensing for the first time and enables harmonized operation across heterogeneous chips by leveraging domain adaptation. Using over 200 h of measurements from nine independent chips, we demonstrate chip‐independent multiplexed biosensing with 99.3% identification accuracy and relative quantification errors of 10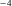 for immunoglobulin G mixtures. Beyond this validation, the platform supports multi‐analyte immunoassays, environmental monitoring, and distributed diagnostics, enabling aligned sensor networks with shared data infrastructures and continuously improving generalization of the common analyzing framework.

## Graphical abstract

We introduce a transformative sensing platform integrating photonic multiplexing with over 10 000 sensors and a deep‐learning framework that combines domain adaptation and cross‐channel fusion for harmonized operation across heterogeneous chips. The platform achieves 99.3% identification accuracy and relative prediction errors of 10 for immunoglobulin G mixtures, enabling multi‐analyte diagnostics, environmental monitoring, and distributed sensing networks.

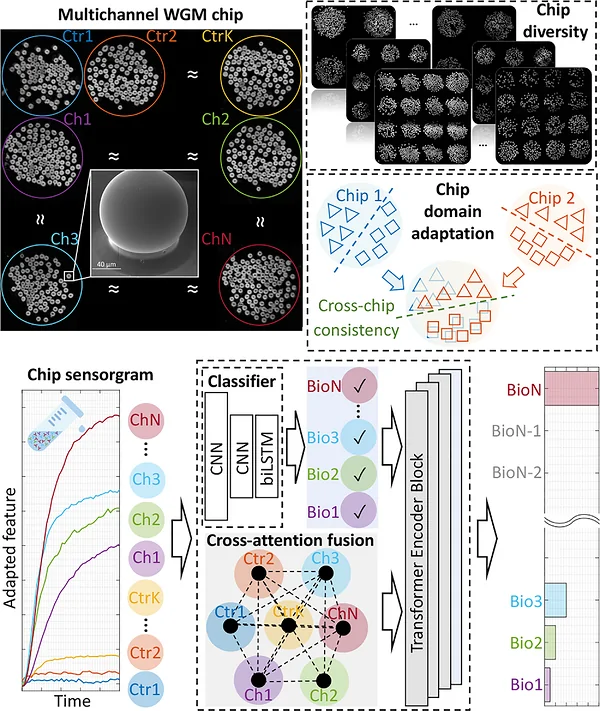

## 1 Introduction

Biosensing is increasingly demanded by personalized medicine and environmental monitoring. Real samples usually contain multiple co‐occurring analytes, which require sensitive, selective and miniaturized multiplexed detectors. For example, simultaneous detection of various immunoglobulins G (IgG) helps in assessing immune status and disease progression. Optical biosensors offer core advantages over labeled assays such as ELISA, including rapid, real‐time, and highly sensitive detection. Whispering gallery mode (WGM) microresonators are highlighted among them by strongly confined optical field and high quality (Q)‐factors, enabling precise real‐time sensing [1, 2, 3, 4]. Perturbations near the cavity, such as refractive index changes, adsorption, or scattering, produce WGM spectral shifts, linewidth changes, or mode splitting [5, 6, 7]. Coupling to plasmonic particles, gain‐assisted/lasing schemes, or exceptional‐point operation further boost WGM performance [8, 9, 10, 11, 12, 13, 14]. Microresonators have already been proven as versatile probes of physical, chemical, and biochemical parameters [9, 15, 16, 17, 18, 19, 20, 21] also as embedded into organisms for intracellular sensing, multiplexed cell tracking, and deep‐tissue imaging [22, 23, 24, 25].

A primary bottleneck in WGM biosensing is scalable multiplexing with dense microresonator integration on a compact, low‐cost platform suitable for point‐of‐care and field diagnostics [26, 27]. Beyond resolving components in complex solutions, multiplexing is crucial for monitoring factors that modulate bioreceptor–analyte affinity such as pH and temperature alongside extensive controls to verify specificity, eliminate false positives, and confirm bioreceptor functionality [28]. These demands make high‐degree multiplexing the core for robust, reproducible biosensing. Although microcavities have a small footprint, their massive interrogation while preserving high Q‐factors and sensitivity remains a major challenge, ultimately dictated by the geometry of the cavities and the light coupling strategy.

Multiplexing with tapered‐fiber bus is achieved by sequentially placing fiber‐melted microspheres in optical contact [29, 30]. This requires nanoscale alignment and exceptional stability (ensured by gluing) [31, 32] and have limited multiplexing due to cavity signal overlapping [33]. Disks, toroids, and goblets on pillars with high Q‐factor improve fabrication scalability, but still rely on external tapered fibers [34, 35] or complex wafer‐integrated fibers [36, 37]. Thus, the complexity drastically grows with cavity count and limits multiplexing to few units [38]. Free‐space coupling is an alternative to fragile tapers for microlasers with performance limited by detector spectral resolution rather than Q‐factor [38, 39, 40]. Recently high Q‐factors have been achieved for free‐space coupling of passive cavities at efficiencies below 0.1% [41], but multiplexing remains limited by sequential cavity scanning. Silicon‐on‐insulator (SOI) rings offer high refractive‐index contrast, CMOS compatibility and scalable fabrication [42, 43], but Si restricts visible‐range operation to more complex ${\mathrm{Si}}_{3}N_{4}$ platforms [44]. Although on‐chip waveguides simplify coupling, Q‐factors rarely exceed $10^{6}$ in air and degrade in aqueous media [45]. Moreover, simultaneous readout demands complex feed waveguide networks often sequentially interrogated by scanning grating couplers [46]. In summary, the integration complexity of known multiplexing schemes linearly scales with resonator number, often operating only in a sequence that fundamentally constrains multiplexing.

A desired real‐time and massively multiplexed sensor inherently generates high‐dimensional data with complex temporal nonlinear correlations, making artificial intelligence and machine learning (AI/ML) analytics essential. Since our first demonstration of ML‐enabled sensing using narrow‐band WGM spectrum [47], the field has progressed toward leveraging high‐dimensional multimode spectra of single cavities [48, 49, 50, 51]. We employed high dimensionality emerging by monitoring hundreds of microresonators at fixed laser wavelength to study static and dynamic processes [52, 53]. Data‐driven models learn extracted informative features and hidden correlations, delivering better sensitivity and broader dynamic range. However, they assume that new measurements match the training data feature space, which ties model to a specific cavity or its fixed assembly. Biosensing datasets span different sensor samples, alignments, and noise conditions, where minor variations among them demand complete retraining, making existing ML approaches unfeasible for field deployment [54]. Furthermore, the high sensitivity of the microresonator makes its high‐Q spectra unique by definition.

We introduce a breakthrough in multiplexed biosensing by synthesising an affordable, modular and densely packaged WGM glass chip with novel hybrid DL‐engine that we named Biological Cross‐Channel Fusion (BioCCF) (Figure 1). The WGM chip is designed to support integration of over ten thousands of cavities with Q $∼10^{6}$ organized into up to 100 independently addressable channels for simultaneous probing, exceeding existing multiplexing capabilities by more than two orders of magnitude and redefining the scalability limits of WGM sensing. Domain adaptation (DA) ensures sensorgram consistency among different chips, whereas DL‐engine joins convolutional–bidirectional LSTM classifier with transformer–encoder regressor enhanced by cross‐attention fusion for fast and accurate prediction of analyte concentrations. Cross‐channel fusion is for the first time employed in biosensing to jointly analyze signals from sensing channels to extract nonlinear correlations between them. BioCCF integrates segments of sensorgrams across the channels, provides robustness to missing or irregular segments and learns correlations among channels, supports scalability by adding new control or sensing channels. Our immunoassay experiments demonstrate prediction on IgG components from complex mixtures in less than 5 min, achieving relative prediction error of $10^{-4}$ for concentrations up to ∼10 μg/ml using nine chips over numerous sensing runs. By eliminating recalibration from chip to chip, the proposed platform establishes a foundation for distributed measurements and harmonized sensing data aggregation from different sites, paving the way toward networked infrastructure for high‐throughput WGM biosensing in real‐world.

**FIGURE 1:**
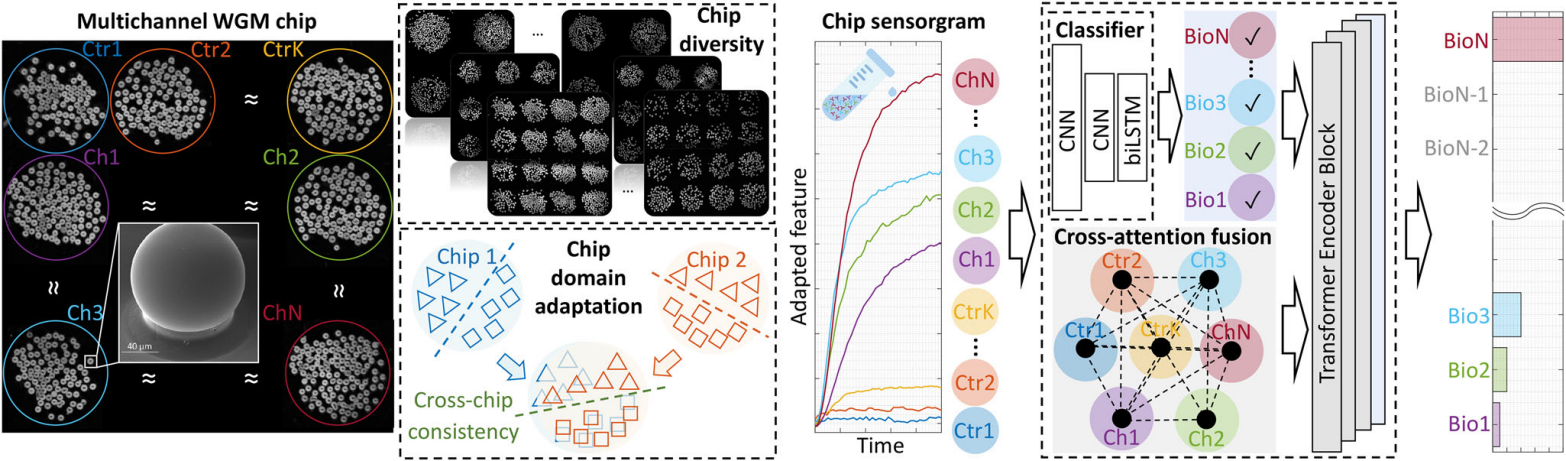
Concept of massively multiplexed biosensing with WGM microresonators. Multichannel WGM chip containing thousands of permanently immobilized glass microspheres organized into N sensing (Ch1–ChN) and K control (Ctr1–CtrK) channels (N+K ≃ 100) and supports simultaneous signal collection. Chip‐to‐chip diversity is mitigated using domain adaptation, enabling consistent sensing responses and facilitating the construction of large aggregated datasets. A hybrid ML‐framework BioCCF combining a CNN/biLSTM‐based classifier with a cross‐attention fusion learns from multichannel chip responses and enables accurate predictions of the individual substance concentrations (Bio1–BioN) in complex solutions.

## 2 Results

### 2.1 Massive WGM Multiplexing

Detecting complex biochemical mixtures requires the number of sensing elements to match or exceed the number of components. We realize this by defining multiple sensing channels on a single glass chip, with microspheres deposited at predetermined locations using 3D‐printed masks containing $n^{2}$ (n integer) pass‐through cells. The microspheres are permanently immobilized by spin‐coating a low‐refractive‐index polymer film (MY‐133MC, MyPolymer). Exemplary chips comprising 4, 9, and 16 sensing channels populated with microspheres from a [106–125] μm batch are shown in Figure 2a–c. For reliable WGM signals determination, cavity maps and cluster assignments are constructed for each chip (Section S1), allowing unambiguous identification of microresonators and their associated channels. Due to the random arrangement and batch‐specific size distribution, the number of cavities varies both across channels and between chips. Statistical analysis of 24 chips shows that increasing the channel count from 4 to 16 reduces the median number of microresonators per channel from 235 to 100, while simultaneously decreasing inter‐chip variance (Figure 2d). Beyond 16 channels, the median cavity count from the batch [106–125] μm drops below 100, limiting sensing reliability (see Domain Adaptation).

**FIGURE 2:**
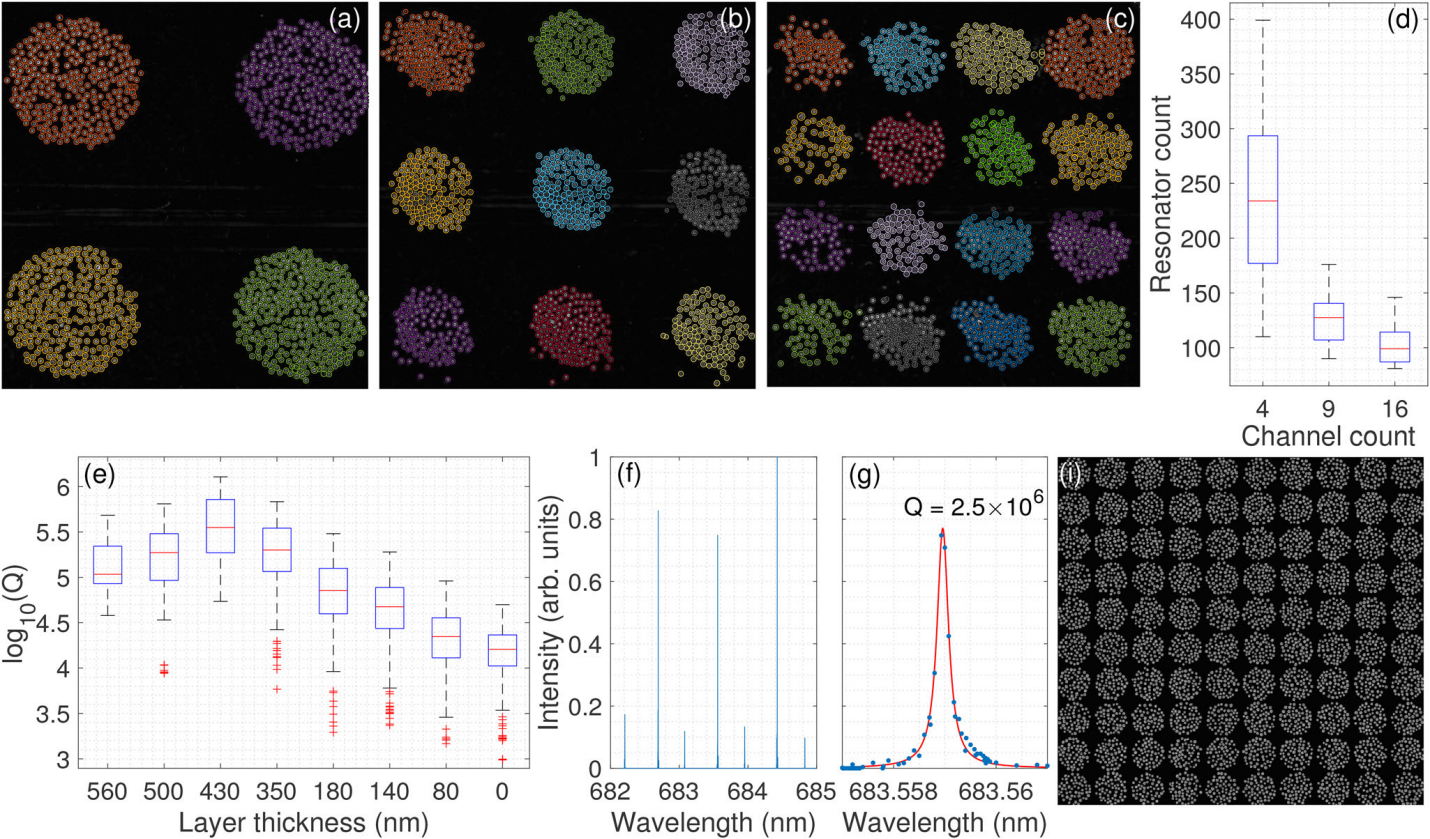
Characterization of the multichannel WGM sensor. (a–c) Exemplary chips with 106:125 μm microspheres allocated in 4 (a), 9 (b) and 16 (c) channels. Each microcavity is channel‐coded with color. (d) Statistics on microresonator filling depending on channel count. (e) Loaded Q‐factor in water fabricated on the preprocessed substrate with distancing layer (80–560 nm) including non‐processed substrate (0). (f) WGM spectrum over 3 nm range of a representative cavity with ≈430 nm distancing layer. (g) Resonance line along with estimations of the Q‐factor achieved by fitting the experimental data (dots) with a Voigt profile (line). (i) Overview of high‐throughput (100 channels) multiplexing with 63:75 μm microspheres.

The fixation layer is critical for signal stability, sensitivity, and sensor reusability being immersed in liquid. Insufficient thickness leads to unstable WGM excitation and poor reusability, whereas excessive thickness increases optical losses and degrades performance. In order to minimize coupling losses we controlled phase matching by the coupling angle, while the field overlap is fine‐tuned by adjusting the prism–microresonator distance set by fixation layer. Numerical calculation, based on the model in Ref. [55], shows that a 100 μm glass microsphere exhibits coupling‐limited Q‐factor ($Q_{coup}$) at $10^{3}$ (680 nm) with zero gap. Increasing the gap to 350 nm raises $Q_{coup}$ to $10^{6}$, and the absorption‐limited value for soda‐lime glass ($Q_{abs}∼10^{7}$) is reached at 470 nm. To achieve a uniform sub‐micrometer spacing, the fixation agent is diluted before spin‐coating. The statistics of loaded Q‐factor over distancing layer thickness is summarized in Figure 2e.

Microresonators without a distancing layer (0 nm) exhibit a median Q of $1.6\times 10^{4}$, with some cavities reaching $5\times 10^{4}$ – well above the numerically predicted $10^{3}$. This discrepancy arises from variance of the cavity sizes and partial embedding in the fixation layer, which prevents full contact with the substrate for smaller spheres. Introducing a distancing layer notably increases the loaded Q, but the contrast of resonances may reduce. At ≈80 nm spacing, over 90% of resonators show clear WGMs, whereas at ≈560 nm less than 5% remain active, while exhibiting Q‐factors of $4\times 10^{4}$‐$5\times 10^{5}$ with a median of $1\times 10^{5}$. The layer of 430 nm provides optimal performance, balancing yield and Q‐factor: resonances are observed for >60% of cavities, with median Q in the mid‐$10^{5}$ range and maxima approaching $3\times 10^{6}$. A representative resonance spectrum and a zoom in the resonance area of $Q>10^{6}$ in aqueous medium are shown in Figure 2f,g. Reducing the microsphere sizes from [106:125] μm to [63:75] μm scales the platform to 100 sensing channels while preserving comparable Q‐factors, with approximately 100 cavities per channel (Figure 2i). This enables high‐throughput operation with up to $10^{4}$ microresonators polled simultaneously.

### 2.2 Domain Adaptation

Despite massive and scalable integration, identical high‐Q WGM chips cannot be produced, leading to strong cross‐chip variability arising from different number of cavities per channel and inherently unique spectra of microresonators. Meaningful sensing therefore requires mapping heterogeneous measurements into a common feature space. Another aspect is the limited experimental throughput that constrains dataset volume and diversity, which must include both technical (same biological sample) and biological (biologically distinct samples) replicates. We address these challenges by converting spectrally‐resolved measurements (acquired via laser sweep) into a unified intensity‐based representation through normalization, spectral and temporal alignment. This transformation generates thousands of effective technical replicates per run, lowering data‐volume constraints. However, spectral uniqueness of microcavities amplifies variability in the intensity domain, hindering direct pattern recognition. We propose domain adaptation to harmonize chip responses via projecting highly heterogeneous data into a shared latent space.

We performed domain adaptation (DA) independently for each single‐wavelength dataset to study the wavelength independence of the channel response in the adapted domain. For each wavelength (≈4800 with a step of 0.2 pm), the number of microresonators (features) is varied from 10 to 200 to assess the consistency of the adapted responses. We also added an ultimate case in which signals from all cavities at all wavelengths are merged as independent features (“Inf”), corresponding in fact to million features/cavities. We randomly split all datasets with different number of features into source and target subsets and repeated the procedure thousand times to ensure statistical robustness. The sensing response is dominated by the spectral shift, which is captured by the first adapted feature (AF1). Higher‐order AFs progressively encode finer spectral variations and ultimately become dominated by noise and local fluctuations. Principle Component Analysis (PCA), Transfer Component Analysis (TCA), Joint Probability Domain Adaptation (JPDA) [56], and Maximum Independence Domain Adaptation (MIDA) with a linear kernel [57] are benchmarked via root‐mean‐square error (RMSE) between the AF1 response and PCA‐derived generalized spectral shift (both normalized). The latter is calculated from the WGM shifts of all microresonators in the channel, each of which being calculated from spectrally resolved data. Benchmark results for different methods and feature number are summarized in Figure 3a–f.

**FIGURE 3:**
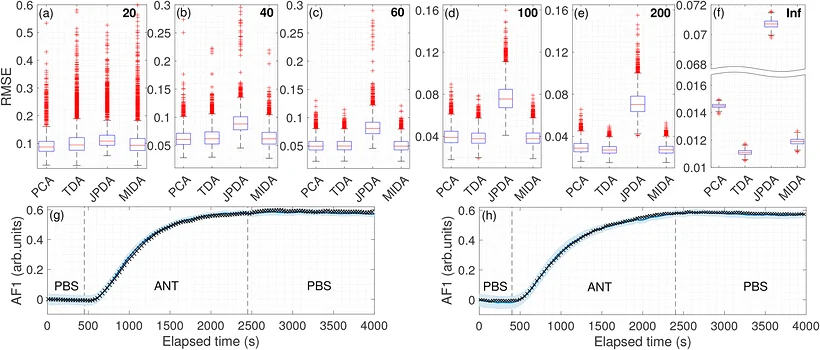
Sensing domain adaptation. (a–e) Consistency of the first adapted feature (AF1) with the generalized spectral shift between wavelengths with varied number of cavities (20–200). (f) Results at infinite (“Inf”) case when all cavities and wavelengths are merged together as separate features. (g,h) Consistency of the domain adaptation procedure with the spectrally‐resolved data processing (black crosses) for two different chips measuring the same biological analyte. Solid blue line shows mean value of the signal in the adapted domain, shaded area ‐ 95% confidence interval.

With fewer than 20 cavities, all adaptation methods yield RMSE values that can exceed 0.5. Increasing cavities number to 60 reduces the median RMSE to ≈0.05 for PCA, TCA, and MIDA, with outliers remaining below 0.15. JPDA performs notably worse, exhibiting a median RMSE of ≈0.07, larger wavelength‐dependent variability, and higher outlier levels. Its performance does not improve even at the infinite number of features. Among the remaining methods, PCA, MIDA, and TCA exhibit progressively better performance with ultimate RMSE = 0.01 with no outliers for TCA. Having 100 microresonators in the channel, TCA achieves a median RMSE of 0.037, a maximum error of ≈0.055 (excluding outliers), and the smallest variance across wavelengths. Selecting 5% deviation threshold as acceptable gives wavelength‐ and cavity‐set‐independent consistency already with 100 cavities in channel. The RMSE will gradually improve toward 0.01 (“Inf” case) with accumulation of chip responses. Having two different physical channels of the same function, the accuracy will be determined as for 200 microcavities, with RMSE = 0.027 and upper deviations <0.04.

The DA procedure aligns the source and target in absolute scale, while agreement with the generalized spectral shift is possible by normalizing the responses. To harmonize signals across chips and channels, AF1 is calibrated after DA. As common in biosensing, the analyte (ANT) dissolved in buffer (PBS) is measured as the sequence of REG‐PBS‐ANT‐PBS phases, where REG denotes the predefined regeneration solution. Owing to the high consistency of the REG–PBS transition, which reflects a bulk refractive index change, the AF1 difference between these two states is used as a calibration reference. Figure 3g,h compares calibrated AF1 and generalized spectral shifts during the ANT–PBS phase for representative measurements (1 μg/mL IgGH, A‐IgGH channels on two different chips). AF1 closely matches the generalized spectral shift in both shape and magnitude, with deviations within confidence interval across different wavelengths. The closely coinciding responses across chips confirm that DA produces robust, chip‐independent dynamical signatures. Summing up, the proposed DA makes the selection of the excitation wavelength irrelevant, eliminating the need for spectral scanning and thus boosting temporal resolution from seconds to milliseconds for true real‐time detection. This significantly improves the flexibility in selection of affordable excitation sources, opening path for distributed accumulation of consistent sensing responses.

### 2.3 Multiplexed Detection

Accurate and reproducible biochemical measurements require stabilization or monitoring of key external parameters, including temperature, pH, incubation time, non‐specific binding and cross‐reactivity. Variations in temperature and pH affect reaction kinetics and binding affinities, while non‐specific adsorption (e.g., electrostatic or hydrophobic interactions) can induce false positives and reduce assay specificity. To ensure uniform reaction conditions, incubation time was fixed at 2000 s and freshly prepared PBS was used to minimize fluctuations in pH and ionic strength. Temperature drift and non‐specific adsorption were actively monitored using dedicated sensing channels: thermal sensing was implemented by isolating a channel from the liquid environment (Section S2), while a channel with bare (non‐functionalized) microspheres served as a reference for non‐specific binding.

We used nine different chips (five with 4, two with 9, and two with 16 channels) each including two functional channels (A‐IgGR, A‐IgGH) and two controls (bare/non‐functionalized and isolated/temperature). For 9 or 16 channel chips, same functions were assigned to multiple channels and their responses were merged. A single sensing run is a PBS–ANT–PBS–REG sequence of phases (2000 s per phase), where IgGH and IgGR solutions, individually and in mixtures, served as analytes (ANT). While some chips were employed as disposable devices, three (one per configuration) were restored and re‐functionalized. Thus, the full data accounts for variations in chip response, regeneration efficiency, and functionalization consistency. So, the experimental design was deliberately limited to four sensing channels, despite the orders‐of‐magnitude higher multiplexing potential of the measurement platform. This approach enabled the establishment of a statistically robust pool of sensing responses, while keeping the experimental timeframe feasible. It also avoids the combinatorial explosion that would result from increasing the number of analytes present in the mixtures concurrently.

The responses exhibiting minimal temperature alterations were selected from the entire dataset to avoid manual signal adjustment using the temperature channel (Section S2). Nonetheless, the entire dataset, including thermally affected measurements, is employed in evaluation of the DL‐model performance later. Two functional and one control channel responses are shown for representative examples of different solutions in Figure 4a–e. They demonstrate two main phases (ANT–PBS), with a prior PBS phase (≈ 400 s) that describes the transport of solution to the sensing chamber. Functional channels indicate responses to their complementary IgG targets, reflecting high specificity, minimal nonspecific adsorption, and negligible cross‐reactivity between IgGH and IgGR. Single‐component solutions yield distinct dynamic responses in the corresponding functional channel only (Figure 4a,b), whereas mixed analytes activate both functional channels (Figure 4c–e). PBS baseline change after analyte binding and its stability indicates durable receptor‐analyte linkage. The bare channel remains inactive, overlapping with the non‐complementary functional channel in single‐analyte and low‐concentration mixtures (Figure 4a–c). At mixtures with higher concentrations of individual components, it exhibits a step‐like response due to bulk refractive index change, which rises with concentration (Figure 4d,e). High analyte concentrations may induce weak signals in non‐complementary channels due to limited stock solution purity (IgGR ≥ 80%, IgGH ≥ 95%). The probability for nonspecific adsorption rises with concentration and partial desorption with signal decay upon PBS exchange may be observed (Figure 4e).

**FIGURE 4:**
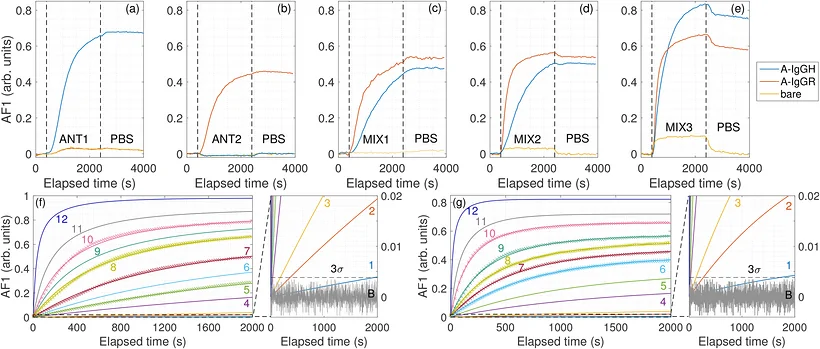
Multiplexed biosensing with optical microresonator chips. (a–e) Multichannel chip responses for single‐ (ANT1: 1 μg/mL IgGH (a), ANT2: 500 ng/mL IgGR (b)) and multi‐component (MIX1: 200 ng/mL IgGH + 1 μg/mL IgGR (c), MIX2: 500 ng/mL IgGH + 2 μg/mL IgGR (d), MIX3: 5 μg/mL IgGH + 5 μg/mL IgGR (e)) solutions. (f,g) Functional channel response consistency in the adapted domain among chips for IgGH (f) and IgGR (g) solutions of various concentrations (PBS (B), 1 ng/mL (1), 5 ng/mL (2), 10 ng/mL (3), 50 ng/mL (4), 100 ng/mL (5), 200 ng/mL (6), 500 ng/mL (7), 1 μg/mL (8), 2 μg/mL (9), 5 μg/mL (10), 10 μg/mL (11), 50 μg/mL (12)). Points represent the measurements with different symbols encoding the measurement repetition and lines ‐ fitted model.

We confined the focus to responses of complementary functional channels for single‐component solutions to evaluate the sensing performance. Each analyte binding phase is fitted with an adapted adsorption model (Section S3), enabling determination of kinetic parameters ($k_{a}$ and $k_{d}$) and detection limits. Experimental data (points) shows strong agreement with the model (lines), yielding RMSE below $3\times 10^{-4}$ and a relative variability in parameters across concentrations under 5%. For IgGR, the mean parameter values are $k_{a}=2.7\times 10^{5}\;M^{-1}s^{-1}$, $k_{d}=3.8\times 10^{-4}s^{-1}$, and $s_{\theta }=1.81$, whereas for IgGH $k_{a}=1.9\times 10^{5}\;M^{-1}s^{-1}$, $k_{d}=1\times 10^{-4}s^{-1}$, and $s_{\theta }=2.04$ (Figure 4f,g). The model remained consistent across the entire concentration range and between measurements using different chips at 0.2 and 1 μg/ml IgGR and 1 μg/ml IgGH (various symbols in Figure 4f,g). This confirms the responses consistency for different chips and acquired at different days. To estimate the detection limit (LOD), we reconstructed the responses for missing concentrations within range (1 ng/mL; 50 μg/mL) and used the approach proposed in [58, 59]. The baseline standard deviation (σ) was obtained for PBS phase, and the sensitivity (S) was derived from a linear fit of signal magnitude at 25 min after start of the binding. Based on LOD=3σ/S, the detection limit of the sensor is estimated as ≈6 pM for both IgGR and IgGH.

### 2.4 Deep‐Learning Processing

Analytical models can describe responses in the adapted domain, but they struggle to incorporate complex, correlated features. For this reason, we considered only single‐component and temperature‐stable responses while fitting (Figure 4). Studying mixtures exhibiting cross‐linking, accounting for temperature or pH‐associated alterations or analyte list expansion would require permanent refinements of the analytical model. Moreover, environmental fluctuations, local perturbations, and missing data introduce noise that substantially degrades analytical accuracy, while the intrinsically nonlinear nature of the sensing responses limits model scalability. Ultimately, this leads to the inability to describe all processes within an analytical model. In contrast, ML architectures naturally accommodate high‐dimensional, nonlinear, and noisy data and improve with increasing data volume, making them a more robust, flexible, and scalable solution for multiplexed biosensing.

Exemplary sensing runs affected by thermal instabilities, each comprising ≈4800 technical replicates, are shown in Figure 5a–c. Temperature fluctuations project coherently across all channels and dominate the responses at low analyte concentrations, particularly in the A‐IgGR (Figure 5b) and A‐IgGH (Figure 5c) channels. For single‐analyte solutions, the bare channel closely follows the non‐complementary functional channel within the 95% confidence interval (Figure 5a,b). The bare channel shows no detectable non‐specific binding and except for high analyte concentrations, its response reflects preliminary temperature variations and exhibits an inverse correlation with the isolated temperature channel (Section S2).

**FIGURE 5:**
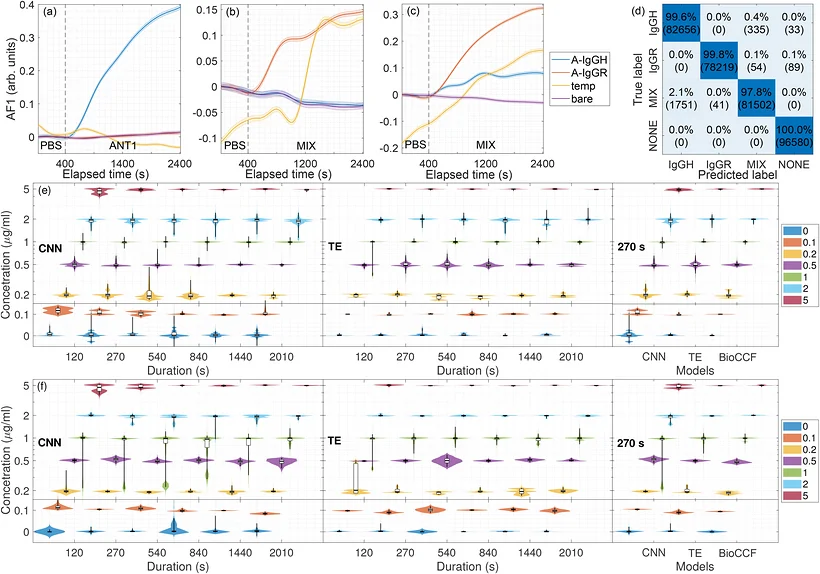
ML‐powered multiplexed biosensing. (a–c) Representative chip sensing runs for the single phase (ANT1: 1 μg/mL IgGH (a), ANT2: 0.1 μg/mL IgGR (b), MIX: 0.1 μg/mL IgGH + 0.5 μg/mL IgGR (c)). The response is calculated over ≈4800 technical replicates (solid line ‐ mean value, shaded area ‐ 95% confidence interval). (d) Confusion matrix for solution composition classification. (e,f) Statistics on prediction accuracies of the individual concentrations for IgGH (e) and IgGR (f) depending on the response duration used in training. CNN, TE and BioCCF‐based ML engines are benchmarked.

Our hybrid DL‐framework BioCCF consists of two units: a classifier to identify the solution composition and regressor that estimates concentrations of individual components (Section S4). Classifier filters out analytes that are not present in the solution, then the predicted classes are used together with the channel responses for regressor training. Given the available dataset, only two concentrations (IgGH and IgHR) compose the BioCCF output, but other biochemical analytes can be integrated by adding corresponding modules without retraining the entire system. To prevent data leakage and ensure realistic accuracy estimates, we used a custom cross‐validation strategy. Each sensing run and all its technical replicates were treated as a set of independent observations. Cross‐validation was performed across all sensing runs, with the number of folds matching them. Each fold uses one sensing run for validation and all remaining for training. The learning procedure was executed independently for each sensing run, and the results were subsequently merged, ensuring unbiased benchmarking on previously unseen data. This strategy allows also to ensure effective independence of the training and validation data, with accounting for the cases where independently prepared biological samples and independently fabricated sensor chips are allocated in the validation and training sets.

Complete dataset, acquired from over 200 h of experimental measurements, comprises 244680 sets of responses including technical replicates. We defined four classes (IgGH, IgGR, MIX and NONE) according to the given dataset, where to reflect the NONE class, the dataset was extended with 96580 chip responses in PBS. In total, the whole data available for training BioCCF network constitutes of 341260 sets of responses, including all biological and technical replicates acquired from 71 independent experiments utilizing 9 independently fabricated chips. Within this set, the number of biological replicates for each concentration of the individual IgG constitute has varied but was not less than 3 and number of the technical replicates for each independent experiment is ≈4800. This dataset contains raw signals from the sensing channels resulting from the domain adaptation procedure, with no prior correction applied for external factors such as temperature alterations or non‐specific molecules adsorption. The tuned architecture of the DL‐framework governs the processing of perturbations to the WGM sensing response caused by external/environmental factors.

Before training BioCCF network, we have excluded first 400 s (pure PBS) from all responses and kept 2000 s of measured responses to improve the DL‐engine performance. The performance of BioCCF classification backbone is summarized in Figure 5d. The confusion matrix shows well balance in the dataset with ≈80 000 responses per class. The classifier achieves an average accuracy of 99.3%, ensuring negligible error propagation to regressor. The NONE class was identified with absolute accuracy and complete separation between single‐component solutions was achieved. The lowest accuracy of 97.8% is identified for MIX samples, which may denote the limited repetitions of all possible mixture combinations.

We benchmarked BioCCF with more common regressor architectures on convolutional neural network (CNN) and Transformer Encoder (TE) (details about architectures in Section S4). While CNN and TE configurations processed channels independently, BioCCF learns interactions between channels that becomes significant with the multiplexing level, particularly for mixed solutions. CNN and TE networks were trained and validated on different durations of sensing responses to determine the shortest one enabling reliable concentration discrimination without overlap. Then, BioCCF has been compared with CNN and TE at optimal response duration.

TE model outperforms the CNN independent on duration. The longer response is available for training, the less is the concentration misclassification rate and the prediction accuracy improves (Figure 5e,f). CNN shows serious misclassification errors, where 1 μg/mL IgGR for up to 1440 s long sequence is predicted as 0.2 μg/mL and partial misclassification for 2 and 0.1 μg/mL IgGH is observed for the full sequence (2000 s). TE‐based model excludes misclassification starting from the 270 s sequence, thus the latter is defined as sufficient to predict the analyte concentrations (taking into account their limited number) and used for training BioCCF model. The misclaffication for BioCCF is also excluded, and the prediction accuracy improves compared to TE. Mean prediction error based upon the median concentration value for IgGH is 35, 8, and 2 ng/ml and for IgGR is 81, 23, and 6 ng/ml for CNN, TE and BioCCF, correspondingly. This indicates the mean values of the absolute concentration uncertainty and means that the cross‐channel fusion allows to improve the prediction accuracy by at least 4 times, so that for the given measurement range the relative prediction error is $10^{-4}$. The variance for individual concentrations decreases for all values except for IgGH 0.2 μg/mL and IgGR 0.5 μg/mL that did not change. The 95% variance range among concentrations demonstrates the experimental variability and improves from 18 to 10 ng/mL for IgGH and from 45 to 14 ng/mL for IgGR solutions, 2 and 4 times, correspondingly. For the baseline and the smallest concentration, the 95% variance range does not exceed 4 ng/ml, where this value can be interpreted as LOD estimated for the BioCCF regression model. Thus, BioCCF effectively handles correlated sensing responses and environmental fluctuations in multiplexed biosensing. Its performance is restricted by the detection limit only, which was quantified using the analytical model fitting to the adapted single‐component responses under strictly controlled experimental conditions (LOD value determined in Section 2.3).

## 3 Conclusion

The combination of the affordable, modular WGM glass chip integrating tens of thousands of high‐Q microresonators across up to hundred parallel sensing channels with a hybrid ML‐engine establishes a transformative platform and a new operational paradigm for high‐throughput multiplexed photonic biosensing. A quantitative benchmark comparing the proposed massively multiplexed WGM biosensing platform with representative state‐of‐the‐art multiplexed photonic biosensing systems demonstrates the advantages of the proposed platform across key performance metrics (Table S1). Domain‐adapted ML‐framework enables chip‐independent, rapid, and accurate concentrations estimations for individual analytes in complex solutions, eliminating the need for chip‐specific recalibration. This approach allows consistent accumulation of sensing data across heterogeneous devices, overcoming fundamental limitations imposed by variability in manufacturing high‐sensitivity devices. Simplicity and affordability of the chip fabrication support disposable sensing formats, mitigating other barriers to practical deployment ‐ the limited stability and reusability of biorecognition layers.

Immunoassay experiments demonstrate prediction of IgG components in complex mixtures within <5 min and with relative prediction error of $10^{-4}$ for up to ∼10 μg/ml concentrations, validated on nine chips over numerous sensing runs. Importantly, this does not represent the ultimate performance capability of the sensor. Optimization of the biorecognition layers, where random immobilization is replaced with controlled receptor orientation, is expected to improve detection limits by orders of magnitude. Alternative strategy is to use artificial antibodies (aptamers) as recognition layers. They have longer lifetime and are robuster to environmental variations, but due to resource‐intensive and time‐consuming sequence selection process are limitedly accessible to the sensing community. By accumulating large, consistent, and independently measured datasets, our sensing platform provides a powerful pathway to accelerate benchmarking, validation and deployment of emerging bioreceptors.

The proposed massive multiplexing approach with cross‐channel fusion strategy is particularly well suited for operation in complex and clinically‐relevant media, as it provides complementary sensing information that assists to account for matrix‐induced interference and interactions between analytes and background components. For this additional control channels, duplication of the sensing channel functions or the placement of competing/complementary bioreceptors for a single sensing target over various groups of microresonators are naturally supported by the chip architecture. Beyond glass microspheres used as elementary sensing units in this study, the platform supports integration of microresonators of different geometries and materials within a single chip. Examples include hybrid integration of glass and PMMA microspheres [20] together with synthesized polymer optical microtodoids [17, 19, 60], enabling extended functionality and multimodal sensing. These microresonators will be treated as additional sensing channels, naturally supported by the scalability of the cross‐channel fusion implemented in the ML‐engine. The latter is transferable to other photonic biosensing technologies that generate sensorgrams, such as recently demonstrated massively multiplexed surface plasmon resonance (SPR) [61]. In this broader context, BioCCF can function as a universal sensing data processing backbone for heterogeneous photonic sensing platforms once data consistency is ensured.

The novel vision of the massive sensor multiplexing opens a pathway for rapid technology expansion and distributed, large‐scale data acquisition across the community. Shared repositories containing diverse sensing datasets, spanning multiple analytes, biorecognition layers, experimental conditions, and laboratories, can continuously enhance the accuracy, robustness, and generalizability of the DL framework. This evolution from isolated instruments to an ecosystem will enable direct sharing of raw biosensing data and establish the foundation for a scalable paradigm in photonic biosensing with real‐world deployment potential.

## 4 Experimental Section

### 4.1 Chemicals

Phosphate‐buffered saline (PBS) pH = 7.4 was prepared in deionized water containing 0.01 M phosphate buffer, 0.0027 M potassium chloride, and 0.137 M sodium chloride. Two immunoglobulin G (IgG) species – rabbit IgG (I8140) and human serum IgG (I2511) – were selected as target analytes (ANT), while rabbit (F9887) and human (F5512) anti‐IgG (A‐IgG) molecules served as bioreceptors enabling selective antibody–antigen binding. Surface functionalization of microcavities employed APTMS ((3‐aminopropyl)trimethoxysilane, 281778) to introduce amino (‐${\mathrm{NH}}_{2}$) groups, followed by antibody immobilization via glutaraldehyde (GA, G7526) cross‐linking. A 1 mM NaOH (S5881) solution was used for sensor regeneration (REG), enabling multiple measurement phases within a single experimental run. All chemicals were obtained from Sigma–Aldrich. Bovine serum albumin (BSA, Thermo Scientific 37525) was diluted to 0.01% and used as a blocking agent to minimize nonspecific adsorption.

### 4.2 WGM Microresonators

Soda‐lime glass microsphere (Cospheric LLC) is the elementary sensing unit of the multiplexed chip. Unlike pure silica, soda–lime glass exhibits better chemical and thermal stability, attributed to the presence of sodium carbonate and calcium carbonate. The majority of microspheres in the supplied batch range in size from 106 to 125 μm. Before chip fabrication, the microspheres undergo cleaning that involves ultrasonication in methanol for 20 min, centrifugation to remove surface contaminants, and drying.

### 4.3 Chip Functionalization

The functionalization process begins with cleaning of the chips containing immobilized microspheres to remove dust and residues remaining after their fabrication. Then chips are treated with low‐pressure oxygen plasma to eliminate organic contaminants and generate surface hydroxyl (–OH) groups. Immediately after activation, vapor phase silanization is performed, which ensures uniform coating and precise control of surface modification compared to the wet‐phase process. The chips are subsequently rinsed with 2‐propanol and ultrapure water to remove unbound silane molecules and dried under nitrogen flow. The silanized chips are processed with 2.5% GA in PBS for 30 min at room temperature and then rinsed with ultrapure water. Channel specificity is ensured by spotting droplets of bioreceptors (A‐IgG) solutions and incubation for 1 h at room temperature to allow immobilization. Finally, the chips are rinsed with PBS to remove unbound receptors and treated with a BSA blocking solution.

### 4.4 Instrument

WGM signals are excited via an optical prism and radiative emission of microresonators is monitored. A monochrome high‐speed global shutter camera (CB262RG‐GP‐X8G3, Ximea) enables spatially resolved acquisition of signals from individual microresonators. Uniform excitation across the chip is ensured by a wide‐field laser beam (≈ 8 mm diameter) collimated with achromatic fiber package (60FC‐T‐4‐M40‐24, Schäfter+Kirchhoff). Beam elongation at the prism interface was compensated by transforming the beam profile from circular to elliptical. With this excitation and detection scheme, plenty of microcavities are excited and tracked at once. The excitation source is a tunable diode laser (Velocity, New Focus; 680–690 nm, 200 kHz linewidth), coupled into a single‐mode fiber. Light polarization is controlled to excite TE modes, and the wavelength is monitored by a wavemeter (WS7‐30, HighFinesse).

WGM chip is mounted onto the prism via immersion oil and assembled with microfluidic flow chamber to construct a sensing head. The reusable chamber, fabricated from polyester ketone ester, contains a single inlet–outlet configuration forming one continuous flow path covering all resonators. A transparent window is incorporated to allow real‐time monitoring of fluid motion and to enable WGM signal collection. Analyte delivery is ensured by a pressure‐driven controller (LINEUP FLOW EZ, Fluigent) combined with a flow‐rate sensor (FLOW UNIT, Fluigent) and multiport valve (M‐SWITCH, Fluigent) to enable sequential injection of solutions.

## Author Contributions

A.S. conceived the idea and designed the project. I.S. and A.S. developed theoretical framework and performed data analysis. A.S. fabricated multiplexed chips and carried out the experiments. I.S., E.T. and A.S. developed data processing methodology. I.S. designed the neural network architecture and implemented the deep‐learning engine. I.S., E.T., A.O., and A.S. contributed to the discussions, theoretical analysis, data validation and interpretation. A.O. provided resources for experimental investigations. A.S. supervised and coordinated the overall research. I.S. and A.S. wrote original draft. All authors discussed the results and contributed to reviewing and editing the manuscript.

## Conflicts of Interest

The authors declare no conflicts of interest.

## Supporting information


**Supporting File**: advs78168‐sup‐0001‐SuppMat.pdf

## Data Availability

The data that support the findings of this study are available from the corresponding author upon reasonable request.
