# Supplementary material for: Massively Multiplexed Photonic Chip With Cross‐Channel Fusion For Harmonized Biosensing
Source: Adv Sci (Weinh). 2026 Oct 8:e78168. Online ahead of print. doi: 10.1002/advs.78168 (PMC13650043; doi:10.1002/advs.78168)
Supplement: Supplementary file 1 [file ADVS-9999-e78168-s001.pdf]

Supplementary Information for:

Massively multiplexed photonic chip with cross-channel fusion for harmonized biosensing

Ivan Saetchnikov[1], Elina Tcherniavskaia[2], Andreas Ostendorf[3], Anton Saetchnikov[3*]

[1]Radio Physics Department, Belarusian State University, Minsk, 220064, Belarus
[2]Physics Department, Belarusian State University, Minsk, 220030, Belarus
[3]Applied Laser Technologies, Ruhr University Bochum, Bochum, 44801, Germany
Corresponding author(s): Anton Saetchnikov. E-mail(s): anton.saetchnikov@rub.de

S1 Microresonator mapping

Due to the random nature for allocation of the microresonators on the chip their individual positions are required to be identified in order to record accurate sensing data. We interpret this as a computer vision problem in which a large number of similarly shaped objects are detected in a 2D image [1]. The approach is based on a pipeline consisting of various preprocessing steps followed by a circle Hough Transform [2] (Fig. S1). Preprocessing steps include image denoising using the BM3D method [3], image sharpening to improve edge visibility, background correction and histogram equalization (Fig. S1 a-d). The preprocessing outcome is a clear image with high contrast between the background and the microresonators and can be directly used as input for circle Hough Transform. A representative chip with corresponding sensor map is shown in Fig. S1 e. The absolute majority of microresonators are identified, only a few remained non- or incorrectly localized. The identification run averaged over >20 chips with optimized parameters delivers the detection accuracy of >97% with minimum of 95% where errors refer to Hough Transform's tendency to underestimate (Fig. S1 f). Among the detected regions the median number of the falsely identified microreresonators (false detection rate, FDR) remains below 3% (Fig. S1 g). The latter are often associated with the microresonators defects, light refractions and obstructive dust particles [4]. Backlighting variations may cause the accuracy of the finely tuned implementation to drop by more than 50%. Therefore, to avoid the need for regular adjustment of the model parameters, consistent backlighting conditions are maintained.

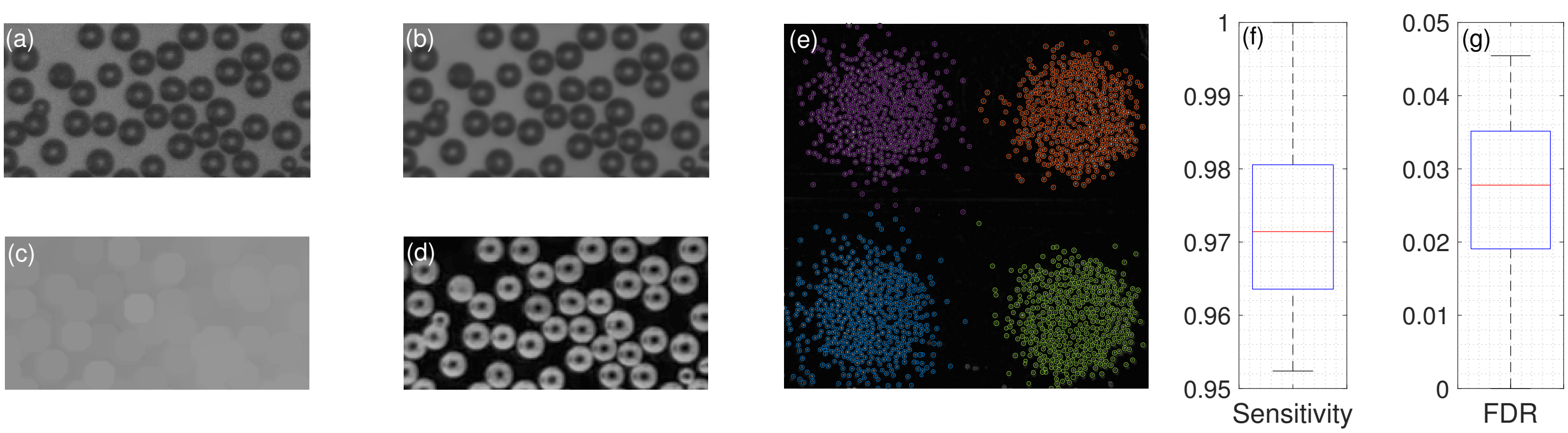

Figure S1: **Microresonator mapping.** (a-d) Multistep preprocessing of the microsphere-based multiplexed chip image. (a) Original image. (b) Denoised image. (c) Estimated background illumination. (d) Final preprocessed image. (e) Overview of identified areas of individual microspheres with color-coded cluster assignment. (f-g) Statistics of the microresonator localization accuracy estimated for >20 multiplexed microresonator chip samples in both sensitivity (f) and FDR (g).

The groups of microresonators acting as sensing channels form a regular $n \times n$ grid on the chip, determined by the geometry of the pass-through mask used for microcavity deposition. Since the number of channels is predefined for each chip and the microresonator positions are known from the previous step, the channel assignment is realized using k-means clustering [5]. The regularity of the cluster locations enables initialization of the cluster centers by uniformly partitioning the chip area into $n \times n$ sections. These predefined cluster counts and their initial centers serve as the input parameters for the k-means procedure. K-means approach is particularly well suited for this task because it minimizes intra-cluster variance and reliably converges to spatially connected clusters, even when initial estimates are approximate. Moreover, its computational efficiency makes it attractive for high-throughput chip characterization. The algorithm exhibits robust performance on chips with accurately positioned microcavities within the clusters. Outlying microresonators that may be accidentally

fixed between the main cluster regions are automatically assigned to the nearest centroid. At the same time, to ensure consistency in the biochemical sensing responses, such outlying cavities are excluded from further analysis.

S2 Temperature control channel

Temperature-induced alterations in the resonator and environmental refractive index (thermo-optical coefficient) as well as resonator physical dimensions (thermal expansion coefficient) disturb the response monitored in single [6, 7] or multiple [8, 9] WGMs. Owing to high coefficient of thermal expansion and negative thermo-optic coefficient, low attenuation loss, good mechanical and chemical stability, polydimethylsiloxane (PDMS) is used for microcavity fabrication [6] or for thin film resonator coating [7] for high-precision thermal sensing. We propose the encapsulation of one of the sensor channels in PDMS by spin-coating to uniformly isolate the cavities from the environment and provide on-chip temperature monitoring. Thus, the mechanism of the thermal response is limited by thermo-optic PDMS changes. PDMS forms a protective hydrophobic layer minimizing interaction with water and reducing non-specific binding of biomolecules. Being transparent in the visible range, it does not hinder signal detection in multiplexed imaging. As long as all microresonators in the channel remain entirely isolated, the remaining inhomogeneity associated with their random distribution does not affect the performance.
The performance of the on-chip temperature channel was evaluated using four sequential PBS–ANT–PBS–REG cycles (2000 s each sensing phase) with 0.1 μg/ml IgGH as the analyte. External temperature sensor (PT 100, resolution 0.01 K) integrated with the sensing head verifies the spectral response consistency of the PDMS-isolated channel with actual temperature fluctuations. The unscaled response of the isolated channel over almost 10 hours, together with PT100 readings, is shown in Fig. S2 a and reveals a clear inverse correlation between the channel response and actual temperature. Only the first phase shows an anomalous positive trend, attributed to thermal balancing between the fluid and sensing head after the assembly. Combining responses from all biochemical phases together confirms a consistent linear dependence between the isolated-channel signal and temperature (Fig. S2 b), as expected for small thermal variations. The residuals around the linear fit primarily reflects PT100 accuracy limits and hysteresis. Since the confidence interval remains nearly constant along the fit, the biochemical medium has negligible influence on the temperature-channel response. Thus, this method reliably captures thermal fluctuations and can be used to correct biochemical signals for temperature-induced drift.

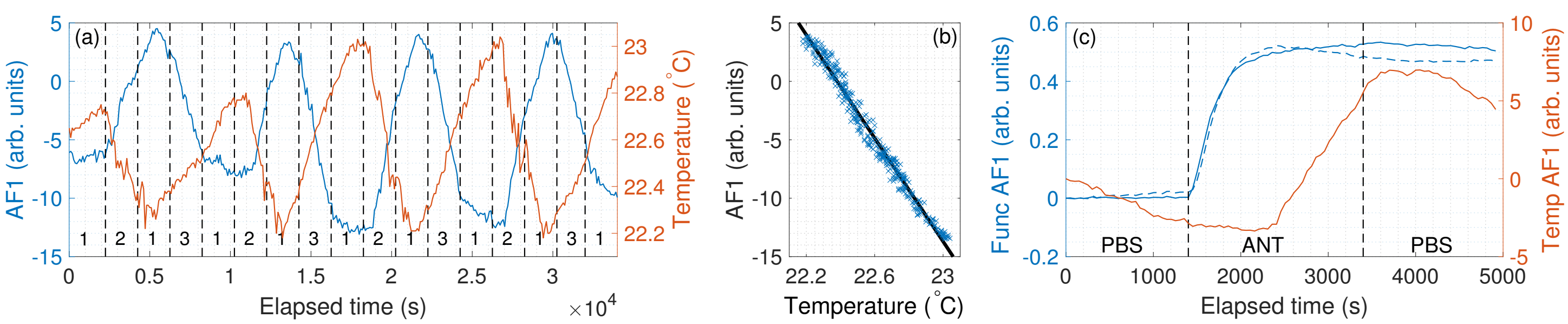

Figure S2: **Temperature control channel.** (a) Spectral response of the PDMS isolated control channel (left axis) along with the temperature variations measured by the external sensor (right axis) in varying biochemical environment (PBS (1), ANT (0.1 μg/ml IgGH) (2) and REG (3)). (b) Linear fit of the channel response to the temperature. (c) The impact of the temperature/isolated channel response (right) for detection of 2 μg/ml IgGR with A-IgGR channel (left) as raw (dashed) and corrected (solid) response curves.

An example of a complementary functional-channel response for a PBS–ANT–PBS sequence, before (dashed) and after (solid) temperature correction, is shown in Fig. S2 c, where the correction has been executed manually under consideration of the fit parameters defined previously. Temperature fluctuations with a total span of 0.8 °C noticeably distort the binding dynamics of a relatively high IgGR concentration (2 μg/ml). This distortion is most evident in the steady-state PBS phases before and after molecular attachment, where correcting for the isolated temperature-channel signal restores temporal consistency. The binding curve itself also becomes more accurate, particularly near saturation, where temperature drift is most pronounced. Overall, incorporating on-chip thermal compensation substantially improves the reliability of functional-channel analysis and enhances sensor performance. Therefore, incorporating the temperature correction function into the ML-engine architecture allows for the development of a robust, flexible, and scalable solution for multiplexed biosensing.

S3 Small molecule adsoprtion model

We used the modification of the small molecule adsorption model that incorporates the surface exclusion effect indicating the capacity of previously adsorbed particles to hinder the adsorption of subsequent molecules [10, 11]. This model is described analytically as follows:

$$\frac{d\theta}{dt} = k_a C \phi(\theta) - k_d \theta, \tag{S1}$$

where θ is the surface coverage, k_a, k_d are the association and dissociation constants and C is the concentration given in mol/L (M) values. Surface exclusion effects are considered by describing the amount of free binding sites via function $\phi(\theta)$ approximated by the empirical expression:

$$\phi(\theta) = \frac{(1-x)^3}{1 - 0.8120x + 0.2236x^2 + 0.0845x^3}, \tag{S2}$$

where $x = \theta/\theta_\infty$ and θ_∞ is equal to 0.547 for spherical particles [10].
To operate with sensing data from the adapted domain, denoted as AF, the scaling parameter has been added to the model. It adjusts the surface coverage $\theta_s = s_\theta \theta$ to sensor response.

S4 BioCCF network model

Novel hybrid ML-framework that we named biological cross-channel fusion (BioCCF) addresses the task of multiplexed analysis of complex biological solutions by combining a classifier for analyte identification with regressor to estimate concentrations of individual analytes (Fig. S3).

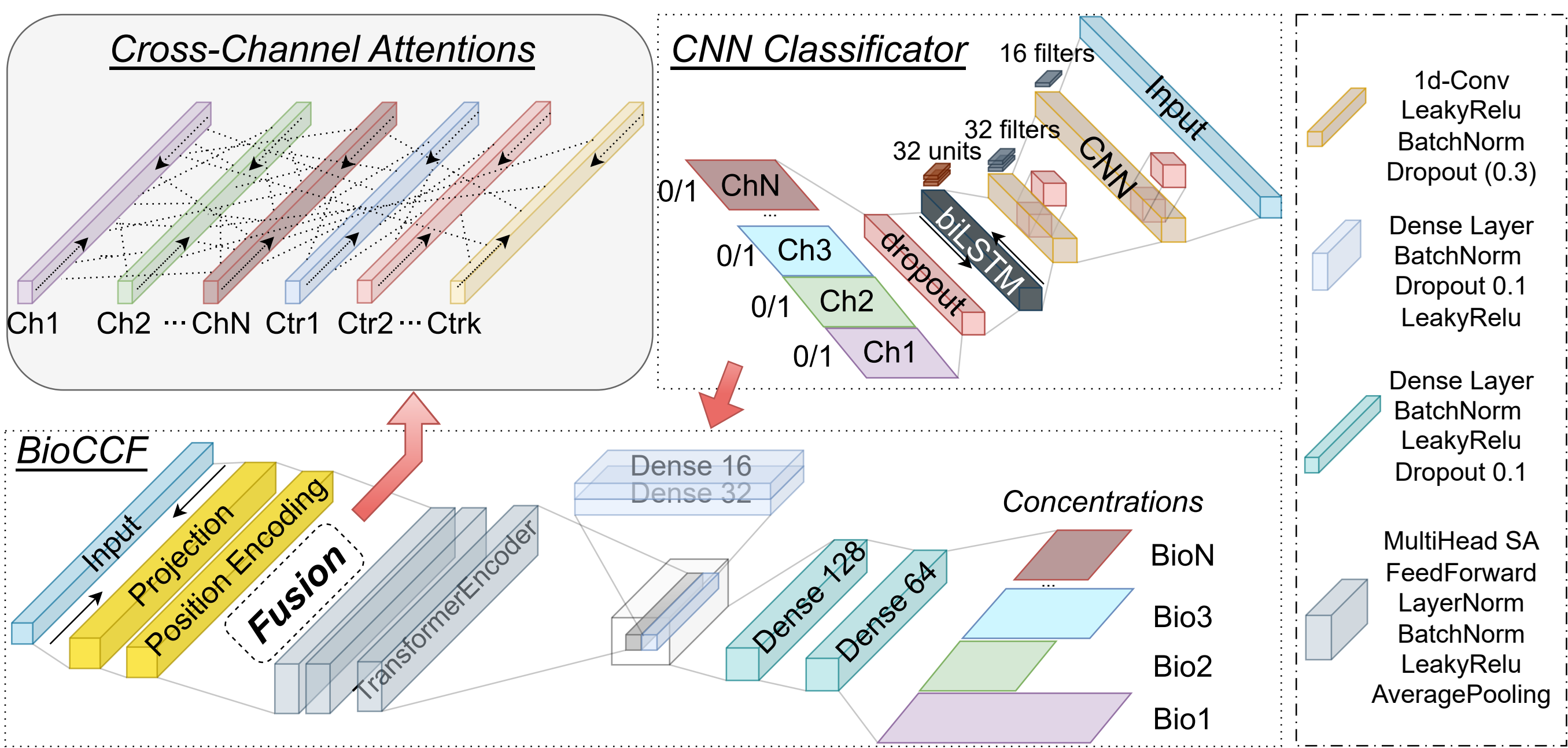

Figure S3: Architecture of the proposed BioCCF network for multiplexed biochemical solutions analysis.

The classification backbone employs a hybrid architecture that integrates convolutional neural network (CNN) layers with bidirectional long short-term memory (BiLSTM) layers. The model includes two sequential 1D convolutional modules with 16 and 32 filters, respectively, and kernel sizes decreasing from 5 to 3 while maintaining consistent padding. Each convolutional block is followed by LeakyReLU activation, batch normalization, and dropout layer with probability of 0.3 to mitigate overfitting. The resulting feature representations are processed with BiLSTM layer out of 32 hidden units, followed by another dropout layer (rate = 0.3) and a final output layer for classification. This design enables the model to first capture local spatial dependencies and suppress noise through convolutional operations followed by learning complex temporal relationships in both forward and backward directions by the BiLSTM component.
The regression backbone is based on Transformer Encoder model where the channel responses are first passed through the projection block and position encoding block combined with cross-feature fusion attention before applying a set of transformer encoders. Projection layer performs a dense transformation of the input into a 16-dimensional space and position encoding generates unique 16-dimensional vector representations for each temporal position in the input. The outputs from the projection layer and position encodings are then passed through the cross-channel fusion. This block is designed to preprocess and learn interactions between different channels. It applies cross-attention to time sections of the channel responses both within single channel and among different channels using a multi-head attention block with 2 heads. The fused outputs then pass through three sequential transformer encoder blocks, each comprising a 4-head attention layer, a feed-forward network with GELU and dropout activations, and a normalization layer. Between the transformer encoder

blocks, batch normalization and LeakyReLU layers are applied to introduce normalization and nonlinearity, alongside average pooling with a kernel size of 2 to reduce sequence lengths. Categorical outputs of the classification backbone are passed through two dense layers of sizes 16 and 32, respectively, each followed by BatchNorm, LeakyReLU, and dropout (0.1). Finally, features extracted from the channel sensorgrams and categorical features are concatenated and passed through two fully connected layers with BatchNorm, LeakyReLU, and dropout, reducing the dimensionality from 128 to 64.
The performance of the novel BioCCF framework for analyzing multiplexed biosensing data was benchmarked in a staged ablation study. We started with a baseline, CNN-based regressor consisting of three modules with 32, 64, and 128 filters (kernel sizes of 5, 5, and 3). Each module was followed by BatchNorm, LeakyReLU, and MaxPooling layers, as well as global average pooling. The CNN regressor does not contain self-attention mechanisms or a cross-channel fusion module; therefore, it serves as a reference model for evaluating the contribution of the proposed architectural advancements. Next, we replaced the CNN modules with three Transformer Encoder (TE) blocks, making self-attention the primary mechanism for temporal aggregation. This modification isolates the contributions of self-attention and long-range temporal tracing, revealing the impact of the Transformer architecture on the regression task. Finally, we extended the TE baseline with a cross-channel fusion module, which enables explicit feature exchange and aggregation across channels. The extracted features were concatenated with additional categorical features provided by the classifier module of the DL framework, then processed according to the BioCCF pipeline. All deep learning models were trained and validated on a hardware with a 14-core Intel Xeon W-2275 processor and 128 GB memory, utilizing NVIDIA Quadro RTX 6000 (24 GB VRAM) GPU to accelerate computation. The development environment was based on Python, employing CUDA for GPU acceleration and leveraging PyTorch as the primary deep learning framework. Data preprocessing, manipulation, and visualization were performed with MATLAB.
The choice of the cross-channel (i.e., cross-feature) fusion is motivated by the physical and biochemical nature of the WGM sensing principle, where meaningful cross-correlations are observed between different sensing channels linked to temperature impact, non-specific adsorption, temporal flow instabilities, and technical variations in the light probe, among others. Alternative fusion strategies based on late fusion with an ensemble of independent models or gated fusion ignore correlated temporal noise of the channels that requires global or pairwise context. With scaling BioCCF to a larger number of functional channels, we anticipate transitioning to a hierarchical cross-channel fusion framework, where strongly correlated channels are first fused locally. These representations are then integrated via a global fusion module before being processed by Transformer Encoder blocks. Thus, such a hierarchical design distributes the computational load and reduces the size of the interaction matrix prior to global fusion processing.

S5 Quantitative benchmarking

A quantitative benchmark comparing the proposed massively multiplexed WGM biosensing platform with representative state-of-the-art systems is presented in Tab. S1. These systems include WGM resonators, silicon/silicon-nitride photonic ring resonators, surface plasmon resonance (SPR) imaging platforms, and other multiplexed photonic biosensing systems. The analysis covers key metrics such as channel and sensing unit numbers, Q-factor, limit of detection (LOD), sensitivity, throughput, scalability, and multiplexing capability.

Table S1: Quantitative benchmarking of representative examples of photonic platforms for multiplexed biosensing (RIU – refractive-index unit; WGM – whispering gallery mode; R6G – Rhodamine 6G, SPR – surface plasmon resonance; ML – machine learning, GMR – guided-mode resonance; PCF – photonic crystal fiber; FBG – fiber Bragg grating; BiMW – bimodal waveguide; GMR – guided mode resonance, quasi-BIC – quasi-bound state in the continuum).

Platform	Photonic architecture	Channels	Sensors	Q-factor	LOD/sensitivity	Throughput	Multiplexing/scalability	Key trade-offs and limitations	Ref.
Passive WGM fiber-tip microsphere	Silica microsphere integrated at a fiber tip; single-mode-fiber coupling with tunable laser interrogation.	2	2	5×10^5	∼6 pg/mm^2 DNA mass loading	10 ms sampling; biological detection within minutes	2 channels in parallel; limited scalability	High sensitivity and fast sampling, but precise fiber coupling/alignment is required. Multiplexing is constrained by spectral overlap and the non-integrated architecture.	[12]
Passive on-chip WGM microtoroid	Silica microtoroid coupled via tapered fiber; frequency-locked laser interrogation.	1	1	10^6	5.5 aM ($\kappa O/DynA$)	Biological detection within 200 s	Multiplexing not demonstrated	Exceptional sensitivity, but tapered-fiber coupling (alignment add system complexity). Multiplexing remains strongly constrained by cavity addressing and spectral overlap.	[13]
Microbubble WGM laser	R6G-doped laser cavity embedded in PMMA fiber; pulsed 532-nm excitation with spectral monitoring.	3	9	4.6×10^5 (simulation)	6.11 aM (IgG); 13.8 pM (IgA); 5.14 pM (IgM); 184.44 nm/RIU	3 min sampling; 8-13 min biological detection	Single bubble demonstrated in parallel	Scaling requires additional fibers/channels and careful spectral separation. The experimental Q-factor is not quantified. Complex system for external excitation and spectral readout.	[14]
Suspended microdisk WGM laser	R6G-doped SU-8 microdisks with free-space/multimode-fiber optical pumping and collection.	1	3	9.8×10^3	17.8 fg/ml (VEGF); 0.3 ng/ml (HRP-streptavidin)	5.5 h biological detection	Multiplexing not demonstrated	High sensitivity, but pumping/readout configuration complicates scaling. Laser-intensity readout is susceptible to pump laser fluctuations.	[15]
Si microring (Genalyte platform)	On-chip silicon microring array with grating-coupled waveguides; free-space optical scanning and tunable laser interrogation referenced to an etalon.	32	136	40,000	60 fM (streptavidin); 7.6×10^{-7} RIU; 163 nm RIU^{-1}	<10 min (anti-SS-A); 8 min (streptavidin)	4 rings (one channel) addressed in parallel; sequential probing of the channels and up to 12 chips possible (1,536 rings)	Strong integration and practical multiplexing density. However, free-space beam steering leads to sequential channel processing rather than fully simultaneous optical readout.	[16, 17]
Si_3N_4 microring	Two Si_3N_4 ring resonators coupled to single bus waveguide; fiber-array probing.	7	14	$> 10^5$	1.5 ng/ml (IL-1β); 3.1 ng/ml (CRP); 7.6 ng/ml (IL-6); 20.7 ng/ml (IL-8)	19 s sampling; 25 min calibration; 2-4 h biological detection	14 rings interrogated in parallel; scalability constrained by fiber-array probing	Compact and highly integrable, but optical interrogation depends on a fiber array. Chip-to-chip and wafer-to-wafer variability was not characterized.	[18]
**Massive WGM multiplexing**	Integrated glass WGM chip, fixed-frequency laser interrogation, parallelized optical readout, and integrated ML processing.	≤100	≳10,000	10^6	6 pM (IgG); classification accuracy (ML) >99%, relative prediction error (ML) $\sim 10^{-4}$	<10 ms sampling; <5 min biological detection	simultaneous addressing ≳10,000 sensors; 4-plex detection (2 IgG + 2 controls) demonstrated	Affordable, modular, and reusable chip architecture with massively parallel sensor readout. Cross-platform and chip-independent ML framework integrated with distributed biosensing supported. Limited probing/readout system integrability.	**This study**
SPR imaging (Horiba Scientific OpenPleX)	Broadband or monochromatic illumination with coupling at fixed angle; reflected-light imaging using a camera.	384	384	N/A	50 nM (anti-Jun); 1.78 nM (anti-p53)	<30 min biological detection	384 spots in parallel; 3-plex detection demonstrated	Excellent spatial multiplexing. Chips are unaffordable, bulky probing/readout system. Limited sensing performance. Sensing data analysis and chip-to-chip variability remain practical considerations.	[19]
Linearly chirped diffraction-grating SPR imaging	Normal-incidence Au diffraction gratings with spatially varying periods; reflected-light imaging.	12	12	N/A	10 pM (miR-221); 19 pM (miR-16); 8×10^{-7}–2×10^{-6} RIU	Real-time sampling; ∼30-35 min biological detection	12 gratings in parallel (8 sensing + 4 reference); scalable by adding gratings; 2-plex detection demonstrated	Compact parallel architecture, but the reported assays requires Au-nanoparticle sandwich amplification. Larger arrays increase grating fabrication and sensing complexity.	[20]
Miniaturized SPR biosensor	Grating-coupled backscattering configuration integrating LEDs, photodiodes, and a nanoplasmonic grating without conventional external coupling optics.	7	7	N/A	3.7 μg/ml (lactoferrin); 10 μg/ml (streptomycin); 10^{-4} RIU	15 min biological detection	7 sensors in parallel; 4-plex detection (2 solutions + 2 references) demonstrated	Very compact and portable, with integrated light sources/detectors and a reusable sensing surface. Increasing channel density can reduce optical signal margin; calibration, cross-reactivity, and data processing remain practical considerations.	[21]
Au-coated PCF with FBGs	Extrinsic plasmonic PCF with FBG-induced cladding-mode resonances and SPR on 40 nm Au layer.	2	2	N/A	1 μg/ml = 8.62 nM (HER2)	<1 min sampling; ∼10-20 min biological detection	2 sensors in parallel; limited scalability	Combines fiber-Bragg-grating and plasmonic responses, but multiplexing density is limited. Fabrication variability requires sensor-specific calibration, while spectral overlap and temperature fluctuations must be managed.	[22]

Continued on next page

WILEY-VCH

Platform	Photonic architecture	Channels	Sensors	Q-factor	LOD/sensitivity	Throughput	Multiplexing/scalability	Key trade-offs and limitations	Ref.
Si_3N_4 waveguide interferometer (BiMW)	One input split into 8 parallel BiMWs using on-chip Y-branch splitters.	8	8	N/A	1.2±0.4 ng/ml (RSV B); $(7 \pm 5) \times 10^{-7}$ RIU	<9 min biological detection	8 sensing elements in parallel (7 sensing + 1 reference)	Demonstrated parallel sensor operation. The architecture requires custom photodetection/readout electronics, and long Y-branch structures increase footprint. Multiplexed detection has not been demonstrated.	[23]
Chirped GMR array	Chirped GMR gratings with spatial spectral encoding; plane-wave LED illumination and camera-based readout.	322	644	N/A	84 nM (WGA); 1 pg/ml (cGMR); 1.63×10^{-3} RIU	6 s sampling	644 optical elements in parallel; 6-plex detection (5 glycopolymers + 1 control) demonstrated	Very high spatial multiplexing with simple imaging readout. Miniaturization and dense packing can impose sensitivity/dynamic-range trade-offs, while sensor-to-sensor variability must be controlled.	[24, 25]
Diatomic quasi-BIC metasurface	Single-frequency laser illumination with transmission imaging and computational signal reconstruction.	12	12	178.6 ± 15.8	204 fM (extracellular vesicles); 315 fM (silica nanoparticles); 305 nm/RIU	14 s sampling	12 sensors in parallel; hundreds projected	High-Q quasi-BIC resonances enable strong field enhancement and spectrometer-free imaging. The platform is sensitive to fabrication and readout noise, requires decoder calibration. Multiplexed detection has not been demonstrated.	[26]

References

[1] A. Saetchnikov, E. Tcherniavskaia, V. Saetchnikov, A. Ostendorf, volume 10678 of *SPIE Proceedings*. **2018** 106780W.

[2] R. O. Duda, P. E. Hart, *Commun. ACM* **1972**, *15*, 1 11–15.

[3] K. Dabov, A. Foi, V. Katkovnik, K. Egiazarian, *Proc. Workshop on Signal Processing with Adaptive Sparse Structured Representations (SPARS'09)* **2009**.

[4] A. Saetchnikov, B. L. Hack, D. Vonk, T. Glasmachers, A. Mosig, A. Ostendorf, volume 12618 of *SPIE Proceedings*. **2023** 1261803.

[5] A. M. Ikotun, A. E. Ezugwu, L. Abualigah, B. Abuhaija, J. Heming, *Information Sciences* **2023**, *622* 178.

[6] C.-H. Dong, L. He, Y.-F. Xiao, V. R. Gaddam, S. K. Ozdemir, Z.-F. Han, G.-C. Guo, L. Yang, *Applied Physics Letters* **2009**, *94*, 23 839.

[7] H. Li, X. Fan, *Applied Physics Letters* **2010**, *97*, 1 011105.

[8] J. Liao, L. Yang, *Light: Science & Applications* **2021**, *10*, 1 32.

[9] H. Li, Y. Lu, S. Zhou, T. Jing, J. Wang, C. Ma, M.-K. Seo, L. Yu, *Optics express* **2024**, *32*, 4 5515.

[10] P. Schaaf, J. Talbot, *The Journal of Chemical Physics* **1989**, *91*, 7 4401.

[11] K. A. Wilson, C. A. Finch, P. Anderson, F. Vollmer, J. J. Hickman, *Biomaterials* **2012**, *33*, 1 225.

[12] F. Vollmer, S. Arnold, D. Braun, I. Teraoka, A. Libchaber, *Biophysical Journal* **2003**, *85*, 3 1974.

[13] A. Gin, P.-D. Nguyen, J. E. Melzer, C. Li, H. Strzelinski, S. B. Liggett, J. Su, *Nature communications* **2024**, *15*, 1 7445.

[14] X. Sun, G. Fang, N. Nie, Z. Yuan, B. Fu, T. Zhou, P.-H. Tseng, Y.-C. Chen, *Laser & Photonics Reviews* **2025**, *19*, 20.

[15] X. Ouyang, T. Liu, Y. Zhang, J. He, Z. He, A. P. Zhang, H.-Y. Tam, *Lab on a chip* **2020**, *20*, 14 2438.

[16] M. Iqbal, M. A. Gleeson, B. Spaugh, F. Tybor, W. G. Gunn, M. Hochberg, T. Baehr-Jones, R. C. Bailey, L. C. Gunn, *IEEE Journal of Selected Topics in Quantum Electronics* **2010**, *16*, 3 654.

[17] S. Mudumba, S. de Alba, R. Romero, C. Cherwien, A. Wu, J. Wang, M. A. Gleeson, M. Iqbal, R. W. Burlingame, *Journal of immunological methods* **2017**, *448* 34.

[18] J. S. Cognetti, M. T. Moen, M. G. Brewer, M. R. Bryan, J. D. Tice, J. L. McGrath, B. L. Miller, *Lab on a chip* **2023**, *23*, 2 239.

[19] C. V. Agu, R. L. Cook, W. Martelly, L. R. Gushgari, M. Mohan, B. Takulapalli, *Communications biology* **2025**, *8*, 1 468.

[20] J. Slabý, M. Bocková, L. Jurgová, K. Chadt, O. Hlavačka, J. Homola, *Sensors and Actuators B: Chemical* **2026**, *453* 139502.

[21] M. Bolognesi, M. Prosa, M. Toerker, L. Lopez Sanchez, M. Wieczorek, C. Giacomelli, E. Benvenuti, P. Pellacani, A. Elferink, A. Morschhauser, L. Sola, F. Damin, M. Chiari, M. Whatton, E. Haenni, D. Kallweit, F. Marabelli, J. Peters, S. Toffanin, *Advanced materials* **2023**, *35*, 26 e2208719.

[22] O. Rusyakina, T. Geernaert, M. Loyez, M. Lobry, K. Chah, P. Mergo, H. Thienpont, C. Caucheteur, F. Berghmans, T. Baghdasaryan, *Sensors and Actuators B: Chemical* **2023**, *382*, 4 133561.

[23] A. Alonso-Fernández, P. Ramírez-Priego, M.-C. Estévez, A. Fernández-Gavela, L. M. Lechuga, *ACS sensors* **2026**, *11*, 7 6071.

[24] A. Kenaan, K. Li, I. Barth, S. Johnson, J. Song, T. F. Krauss, *Biosensors & bioelectronics* **2020**, *153* 112047.

[25] L. M. Miller, C. P. Reardon, K. G. Leslie, C. D. Silver, J. S. Male, C. S. Mahon, T. F. Krauss, S. Johnson, *Biosensors & bioelectronics* **2026**, *305* 118669.

[26] Y. Jahani, E. R. Arvelo, F. Yesilkoy, K. Koshelev, C. Cianciaruso, M. de Palma, Y. Kivshar, H. Altug, *Nature communications* **2021**, *12*, 1 3246.
